# Link Connectivity and Coverage of Underwater Cognitive Acoustic Networks under Spectrum Constraint

**DOI:** 10.3390/s17122839

**Published:** 2017-12-07

**Authors:** Qiu Wang, Hong-Ning Dai, Chak Fong Cheang, Hao Wang

**Affiliations:** 1 Faculty of Information Technology, Macau University of Science and Technology, Macau, China; qiu_wang@foxmail.com(Q.W.); cfcheang@must.edu.mo (C.F.C.); 2 Norwegian University of Science and Technology, 6009 Aalesund, Norway; hawa@ntnu.no

**Keywords:** connectivity, coverage, underwater acoustic networks, cognitive radio, spectrum availability

## Abstract

Extensive attention has been given to the use of cognitive radio technology in underwater acoustic networks since the acoustic spectrum became scarce due to the proliferation of human aquatic activities. Most of the recent studies on underwater cognitive acoustic networks (UCANs) mainly focus on spectrum management or protocol design. Few efforts have addressed the quality-of-service (QoS) of UCANs. In UCANs, secondary users (SUs) have lower priority to use acoustic spectrum than primary users (PUs) with higher priority to access spectrum. As a result, the QoS of SUs is difficult to ensure in UCANs. This paper proposes an analytical model to investigate the link connectivity and the probability of coverage of SUs in UCANs. In particular, this model takes both topological connectivity and spectrum availability into account, though spectrum availability has been ignored in most recent studies. We conduct extensive simulations to evaluate the effectiveness and the accuracy of our proposed model. Simulation results show that our proposed model is quite accurate. Besides, our results also imply that the link connectivity and the probability of coverage of SUs heavily depend on both the underwater acoustic channel conditions and the activities of PUs.

## 1. Introduction

There is a growing interest in exploring the underwater environment due to a diversity of underwater applications, including ocean environment monitoring, offshore structural health monitoring, mine reconnaissance, distributed tactical surveillance, target tracking, shipping safety, and fish farm monitoring [1,2,3]. A key technology to enable various underwater monitoring applications is underwater sensor networks (USNs), which can be deployed in various underwater environments such as reservoirs, lakes, rivers, and oceans. Due to the high attenuation of electromagnetic waves in underwater environments, acoustic communications are typically used in USNs.

However, the underwater acoustic spectrum is becoming a scarce resource for the following reasons: (1) the high competition for acoustic spectrum between different users due to the proliferation of man-made underwater acoustic systems, including sonars, acoustic sensors, and unmanned underwater vehicles [4]; (2) the co-existence of *man-made acoustic systems and natural acoustic systems* (e.g., marine animals like whales, dolphins, and sea lions) [1]. It is worth mentioning that we often ignore the interference caused by man-made acoustic systems to marine animals that may use the same frequencies as the human-made acoustic systems. Therefore, it is necessary to solve the spectrum scarcity problem in an environmentally-friendly and spectrum-efficient way.

Cognitive acoustic communications are one of the most promising solutions to the acoustic spectrum scarcity problem. We name such underwater cognitive acoustic sensor networks as UCANs. In UCANs, there are two types of acoustic spectrum users: primary users (PUs) and secondary users (SUs). PUs (including both artificial and natural acoustic systems) have the higher priority to use the acoustic spectrum. SUs (usually only include artificial acoustic systems) have a lower priority to use acoustic spectrum only when the acoustic spectrum is idle or their communications do not hamper the communications of PUs; the awareness of the existence of PUs can be achieved by using spectrum-detecting technology [5]. In fact, cognitive radio technology has been well investigated in terrestrial wireless networks. However, underwater acoustic communications are significantly different from terrestrial wireless communication systems due to the unique features of underwater acoustic channels (e.g., frequency-dependent attenuation and long propagation delay). Therefore, UCANs bring new research challenges in investigating cognitive radio technology in underwater environments.

Cognitive acoustic communications have recently received substantial attention. Luo et al. [1] summarized the research challenges in underwater cognitive acoustic networks. A dynamic spectrum-borrowing algorithm was proposed in [6]. Most recently, a receiver-initiated spectrum management system for underwater acoustic networks was developed in [7]. However, few studies focus on the quality-of-service (QoS) of SUs in UCANs.

Essentially, SUs are spectrum-constrained in UCANs compared with PUs who have a greater chance to access acoustic spectrum than SUs. Therefore, it is more difficult to guarantee the QoS of SUs than that of PUs. One of the most important QoS metrics is the link connectivity and the coverage probability; the former concerns the possibility of whether a pair of nodes can establish a communication link, and the latter concerns the probability that a user is able to achieve a certain threshold signal-to-noise ratio (SNR). *To the best of our knowledge, there is no study on the link connectivity and coverage probability of SUs in underwater cognitive acoustic networks.*

The main goal of this paper is to investigate the link connectivity and coverage of SUs in UCANs. It is non-trivial to analyze the link connectivity and the coverage probability of SUs because of the influence of various ambient factors caused by the complicated underwater acoustic channel and the activities of PUs. The major research contributions can be summarized as follows.

- We develop a novel analytical model to investigate the link connectivity and the coverage probability of SUs in UCANs. We find that the link connectivity and coverage of SUs depends on both the spectrum availability and the topological connectivity, while the spectrum availability has been ignored in most existing works.
- We conduct extensive simulations to verify the accuracy of our proposed model. The simulation results match the analytical results, implying that our proposed model is fairly accurate.
- We observe that the probability of connectivity and the probability of coverage are affected by acoustic signal frequency, various ambient factors (spreading factor and wind speed), and the activity of PUs. Our results also offer some useful insights in designing QoS-aware UCANs.

The rest of this paper is organized as follows. We summarize related works in Section 2. Section 3 presents the system model of UCANs. We analyze the link connectivity and the probability of coverage of SUs in UCANs in Section 4. Section 5 gives the simulation results, considering various factors. Finally, we conclude the paper in Section 6.

## 2. Related Works

There is a growth of activities exploring aquatic environments such as ponds, lakes, rivers, and oceans. An underwater sensor network (USN) is an important technology enabling us to monitor various underwater environments [8,9,10,11,12,13]. USNs consist of sensor nodes that are connected through wired or wireless manners. Compared with wired networks, wireless connection of sensor nodes can greatly reduce the deployment cost and offer the flexibility to different environments [14]. Due to the high attenuation of EM waves in underwater environments, acoustic communications are more scalable in various aquatic environments due to the high penetration of acoustic signals and the long communication range.

There is a growing interest in investigating cognitive radio technology in UASNs since the acoustic spectrum has become a scarce resource due to the proliferation of human aquatic activities. Baldo et al. proposed a channel allocation mechanism to efficiently manage acoustic spectrum in UASNs in [15]. A spectrum signaling approach was proposed for distributed channel allocation for the underwater acoustic networks in [16]. Bicen et al. proposed a dynamic spectrum sharing mechanism in cognitive radio (in terrestrial wireless networks) in underwater acoustic networks in [17]. In [1], research challenges in applying cognitive radio technology in UASNs are summarized. Meanwhile, a spectrum management mechansism with the integration of physical layer schemes and Media Access Control (MAC) layer schemes was proposed in [4]. Moreover, Wang et al. proposed a dynamic spectrum borrowing algorithm in [6]. In [18], a novel statistical power adaptation over multiple-input and multiple-output (MIMO) generalized frequency division multiplexing (GFDM) was proposed for underwater acoustic networks. Luo et al. developed a receiver-initiated spectrum management system for underwater acoustic networks in [7]. In [5], a spectrum detecting scheme was developed to avoid the interference to PUs (especially for marine animals like dolphins).

However, few studies have concentrated on quality-of-service (QoS) in cognitive acoustic underwater networks. Since PUs have higher priority to access acoustic spectrum than SUs, the QoS of PUs is often guaranteed. In contrast, SUs are spectrum-constrained. Therefore, the QoS of SUs is difficult to ensure, which nevertheless has been ignored in most of studies on cognitive acoustic underwater networks. Therefore, the goal of this paper is to investigate two of the most important QoS metrics—the link connectivity and the probability of coverage.

## 3. System Model

Herein, we first give the problem definition in Section 3.1. Then, we introduce the network model in Section 3.2, and the underwater channel model in Section 3.3.

### 3.1. Problem Definition

**Figure 1:**
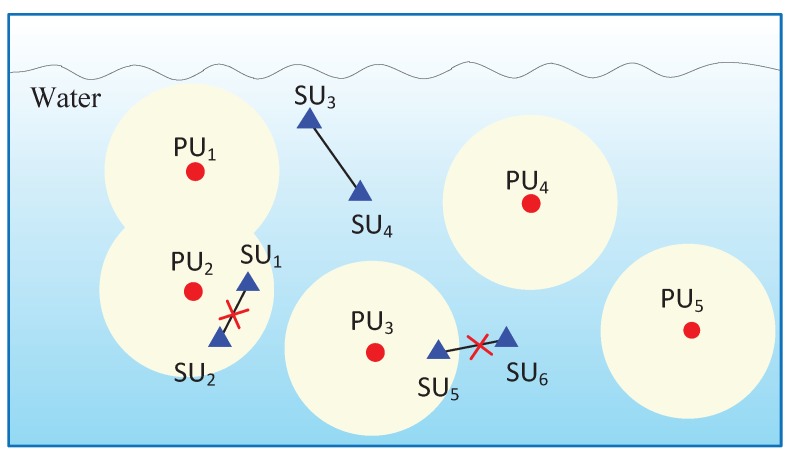
Underwater network model. PU: primary user; SU: secondary user.

Figure 1 presents an example of UCANs, in which primary users (PUs) denoted by red circles can be natural acoustic systems (e.g., marine mammals) or man-made acoustic communication systems (e.g., underwater sensors), while secondary users (SUs) denoted by blue triangles can be cognitive acoustic communication nodes. SUs can transmit data only when their transmissions do not hamper the communications of PUs. Therefore, some SUs must be silent if there are PUs close to them. For example, as shown in Figure 1, SU_5_ cannot establish a communication link with SU_6_ due to the possible interference to PU_3_, as PU_3_ is in close proximity to SU_5_. Similarly, SU_1_ and SU_2_ cannot connect with each other successfully because of the existence of PU_2_, which is close to both SU_1_ and SU_2_. As illustrated in this typical UCAN, we observe that the link connectivity of SU pairs is more difficult to ensure that of PUs, and consequently SU pairs have a lower probability of coverage. Therefore, we aim to investigate the link connectivity and the probability of coverage of SUs in UCANs in this paper.

### 3.2. Network Model

In our network model, we assume that PUs are distributed according to homogeneous Poisson point process (HPPPs) with intensity $\lambda_{p}$ [19]. Each PU radiates the identical power, denoted by $P_{p}$. Since we are concerned with the link connectivity and the probability of coverage of an SU pair, we consider that this SU pair is located at the center of this network within a 2-D plane; this assumption is consistent with the sophisticated stochastic geometric method proposed in [20]. We consider that a secondary transmitter sends a packet, with the power denoted by $P_{s}$.

### 3.3. Channel Model

In the underwater environment, acoustic signal experiences both attenuation and ambient noise. We adopt a classic channel model first presented in [21]. This model has been widely used in most previous studies, such as [5,22,23,24,25,26,27,28]. In order to ensure that the paper is self-contained, we present the channel model including the attenuation component in Appendix A and the ambient noise component in Appendix B.

## 4. Analysis of Link Connectivity and Probability of Coverage

In this paper, we evaluate the link connectivity of SUs in UCANs by probability of connection, which is defined as follows:

**Definition** **1.** Probability of connection is the probability that two SUs can successfully establish a bidirectional link.

It is worth mentioning that we consider a bidirectional link in this paper because it is more general than previous works, as most communications between a transmitter and a receiver require an acknowledgement (i.e., ACK) [4,7].

In UCANs, two SUs can successfully establish a bidirectional link if and only if the following two conditions are satisfied:

(1) Both of the SUs can connect topologically;
(2) Both of the SUs have the spectrum.

Essentially, condition (1) requires that the transmission range of each SU covers each other, and condition (2) requires each SU to obtain the spectrum. Therefore, we first analyse the topological connection condition in Section 4.1. We then derive the probability that two SUs have the spectrum in Section 4.2. We next obtain the probability of connection of two SUs in Section 4.3. Finally, we analyze the probability of coverage in Section 4.4.

### 4.1. Topological Connection Condition

We define the probability that two SUs can topologically connect each other by $p_{top}$, which can be expressed as

(1) $$p_{top}=P\left[SNR_{dB}\geq \delta_{s}\right],$$

where $SNR_{dB}$ is the signal-to-noise ratio in dB between a pair of SUs, and $\delta_{s}$ is the threshold that a receiver can successfully received information.

The signal-to-noise ratio between a pair of SUs, denoted by SNR, can be expressed as follows:

(2) $$SNR=\frac{P_{s}}{\int_{B}A\left(r,f\right)df\int_{B}N\left(f\right)df},$$

where *B* represents the bandwidth used in UCANs. Since we are concerned with the relationship between frequency *f* and SNR, we normalize the bandwidth. Thus, Equation (2) can be simplified to

(3) $$SNR=\frac{P_{s}}{A(r,f)N(f)}.$$

Then, $SNR_{dB}$ can be expressed as

(4) $$SNR_{dB}=10\log P_{s}-10\log A\left(r,f\right)-10\log N\left(f\right).$$

Combining Equation (4) and Equation (A2) (as defined in Appendix A), we can have $p_{top}$ as follows,

(5) $$p_{top}=P\left[k\cdot 10\log r+r\cdot 10\log\alpha \left(f\right)+10\log N\left(f\right)\leq 10\log P_{s}-\delta_{s}\right].$$

**Figure 2:**
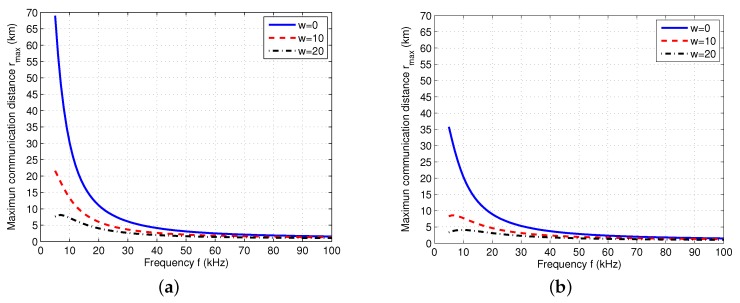
Maximum communication distance $r_{\max}$ (km) with different spreading factor *k* and wind speed *w* (m/s) when s=1, $P_{s}=100$ dB, and $\delta_{s}=20$ dB. (**a**) k=1; (**b**) k=2.

We can see that the left-hand-side (LHS) of the inequality in Equation (5) is an increasing function of the distance *r*. If we let LHS of the inequality be equal to RHS, we can have the *maximum communication distance*$r_{\max}$. However, since the inequality is a transcendental function of *r*, it is impossible to get a closed-form expression of $r_{\max}$. Therefore, we calculate the numerical results of $r_{\max}$. After setting $\delta_{s}=20$ dB [29] and $P_{p}=110$ dB, we obtain the numerical results of $r_{\max}$ with different frequency *f*, spreading factor *k*, and wind speed *w*, as shown in Figure 2. From Figure 2, we can see that $r_{\max}$ varies with *f*. In particular, a higher value of *f* leads to a lower value of $r_{\max}$. Moreover, a higher wind speed *w* results in a lower $r_{\max}$. This is because a higher wind speed brings higher noise according to the underwater acoustic channel model. Furthermore, if we compare Figure 2a with Figure 2b, we can find that $r_{\max}$ decreases with the increased spreading factor *k*.

According to the above analysis, we have $p_{top}$ given as follows,

(6) $$p_{top}=\left\{\begin{array}{cc} 1 & r\leq r_{\max}\\ 0 & r>r_{\max} \end{array}\right..$$

### 4.2. Spectrum Availability

Next, we analyze the spectrum availability of a pair of SUs. In UCANs, in order to avoid interference from SUs to PUs, a spectrum sensing process has to be conducted at SUs before transmissions are initiated [5,30]. During the spectrum sensing process, we assume that if the SNR (dB) at an SU is no less than a detection threshold $\delta_{d}$ (dB), the SU must be silent (i.e., it cannot transmit data). In other words, an SU cannot have spectrum if the following condition is satisfied:

(7) $$SNR_{dB}=10\log P_{p}-10\log A\left(r,f\right)-10\log N\left(f\right)\geq \delta_{d}.$$

**Figure 3:**
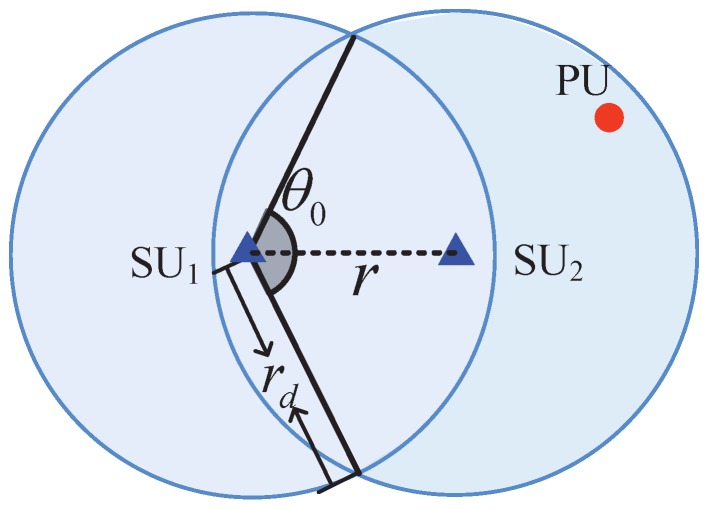
Detection region of a pair of SUs.

Similar to the calculation of $r_{\max}$, if we let LHS of Equation (7) be equal to RHS and combine with Equations (A2)–(A5) (defined in Appendix A and Appendix B) and Equation (7), we can then obtain the *detection range* of an SU, which is defined as the maximum distance that an SU can detect PUs, denoted by $r_{d}$. Similar to $r_{\max}$, we can obtain the numerical values of $r_{d}$. In particular, Figure 3 shows a detection region of a pair of SUs (e.g., SU_1_ and SU_2_). As shown in Figure 3, the detection region of each SU is a circle with a radius of *detection range* $r_{d}$. We observe that a pair of SUs can have the spectrum if both of the following conditions are satisfied:

(1) No PUs in the detection region of SU_1_;
(2) No PUs in the detection region of SU_2_.

According to the fact that PUs follow HPPP, we can have the probability that two SUs can have the spectrum, denoted by $p_{spe}$, as follows:

(8) $$p_{spe}=e^{-S\lambda_{p}},$$

where *S* is the area of the detection region of two SUs, depicted as the blue region as shown in Figure 3. We can see from Figure 3 that *S* is a piecewise function of the distance *r*. Then, *S* can be expressed as:

(9) $$S=\left\{\begin{array}{cc} \pi r_{d}^{2}\;+\;\left(\pi -\theta_{0}\right)r_{d}^{2}\;+\;r_{d}\sin\left(\frac{\theta_{0}}{2}\right)r & r\leq 2r_{d}\\ 2\pi r_{d}^{2} & r>2r_{d} \end{array}\right.,$$

where $\theta_{0}=2\arccos\frac{r}{2r_{d}}$.

After combining Equation (9) with Equation (8), we can have $p_{spe}$ as follows:

(10) $$p_{spe}=\left\{\begin{array}{cc} e^{\left(\pi r_{d}^{2}\;+\;\left(\pi \;-\;\theta_{0}\right)r_{d}^{2}\;+\;r_{d}\sin\left(\frac{\theta_{0}}{2}\right)r\right)\lambda_{p}} & r\leq 2r_{d}\\ e^{2\pi r_{d}^{2}\lambda_{p}} & r>2r_{d} \end{array}\right..$$

### 4.3. Link Connectivity

We denote the probability of connection of an SU pair by $p_{con}$. According to the above analysis of the maximum communication distance $r_{\max}$ in Section 4.1 and the probability that a pair of SUs have the spectrum $p_{spe}$ in Section 4.2, we have the $p_{con}$ as follows:

(11) $$p_{con}=\left\{\begin{array}{cc} p_{spe} & r\leq r_{\max}\\ 0 & r>r_{\max} \end{array}\right..$$

Combining Equation (11) with Equation (10), we can obtain $p_{con}$ if $2r_{d}<r_{\max}$ as follows:

(12) $$p_{con}=p_{top}\cdot p_{spe}=\left\{\begin{array}{cc} e^{\left(\pi r_{d}^{2}\;+\;\left(\pi \;-\;\theta_{0}\right)r_{d}^{2}\;+\;r_{d}\sin\left(\frac{\theta_{0}}{2}\right)r\right)\lambda_{p}} & r\leq 2r_{d}\\ e^{2\pi r_{d}^{2}\lambda_{p}} & 2r_{d}<r\leq r_{\max}\\ 0 & r>r_{\max} \end{array}\right..$$

If $2r_{d}>r_{\max}$, $p_{con}$ can be expressed as:

(13) $$p_{con}=\left\{\begin{array}{cc} e^{\left(\pi r_{d}^{2}\;+\;\left(\pi \;-\;\theta_{0}\right)r_{d}^{2}\;+\;r_{d}\sin\left(\frac{\theta_{0}}{2}\right)r\right)\lambda_{p}} & r\leq r_{\max}\\ 0 & r>r_{\max} \end{array}\right..$$

### 4.4. Probability of Coverage

In traditional terrestrial cellular networks, the probability of coverage is defined as the probability that the received signal-to-interference-plus-noise ratio (SINR) is higher than a threshold [20]. Specifically, for cellular networks with a distribution of distance between a user and a base station, the probability of coverage is the expectation (in terms of the distance between a user and a base station) of the probability that the received SINR is higher than a threshold [20]. However, for SUs in UCANs, if signal from an SU can cover another SU (i.e., an SU can connect with another SU), it needs two conditions: (1) SUs can topologically connect to each other (refer to Section 4.1); (2) both SUs have the spectrum (refer to Section 4.2). Therefore, in contrast to terrestrial cellular networks, we originally define the probability of coverage of SUs in UCANs as the expectation (in terms of the distance between two SUs) of the probability that an SU can connect with another SU. Then, the probability of coverage, denoted by $p_{cov}$, can be expressed as follows:

(14) $$p_{cov}=E_{r}\left[p_{con}\right],$$

where $E_{r}\left[*\right]$ is the expectation of ∗ in terms of the distance *r*.

We assume that the distance between a pair of SUs *r* follows the uniform distribution in $\left(0,R_{d}\right]$, where $R_{d}$ is the maximum distance of *r*. Then, Equation (14) can be expressed by

(15) $$p_{cov}=\int_{0}^{R_{d}}p_{con}\cdot f_{r}\left(r\right)dr=\int_{0}^{R_{d}}p_{con}\frac{1}{R_{d}}dr,$$

where $f_{r}\left(r\right)$ is the probability distribution function (PDF) of *r* and $p_{con}$ is given by Equations (12) and (13).

## 5. Simulations

In this section, we conducted extensive simulations to evaluate the accuracy and the effectiveness of our proposed analytical model on the link connectivity and the probability of coverage in UCANs. In particular, we describe the simulation method in Section 5.1. We then present the simulation results on the link connectivity and the probability of coverage in Section 5.2 and Section 5.3, respectively. Finally, we discuss the implications of our results and point out the future directions in Section 5.4.

### 5.1. Simulation Method

**Figure 4:**
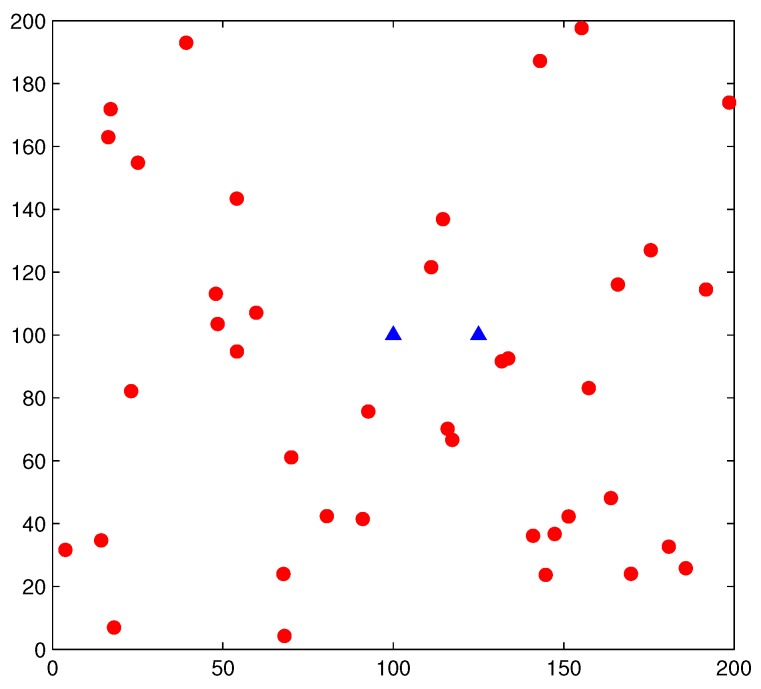
Random topology of a simulation snapshot of underwater cognitive acoustic sensor networks (UCANs), where red circles denote PUs and blue triangles denote SUs.

When we conducted this simulation-based study to evaluate the accuracy of our proposed models, it was necessary to conduct extensive simulations. In particular, PUs are distributed according to HPPP in a plane of area 200 × 200 km^2^. Figure 4 shows a random topology of a simulation snapshot, where red circles denote PUs, and blue triangles denote SUs (the distance between a pair of SUs is *r*). Then, the probability of connectivity $p_{con}^{s}$ of simulations can be acquired by

(16) $$p_{con}^{s}=\frac{\#\;\text{topologies that an SU pair can connect successfully}}{\Omega },$$

where # denotes “the number of”. Note that we denote the simulation result by $p_{con}^{s}$ in order to differentiate it from the analytical one. Once system parameters are chosen, we will repeat the same experiment with different random topology of PUs for Ω times. As indicated in Bettstetter’s seminar work [31], to obtain an approximated result to the analytical one, we need to choose a large enough Ω (theoretically Ω→∞). However, it is extremely time-consuming to obtain such results. In this paper, we choose Ω = 20,000 (which was shown to be large enough in a recent work [32]). More specifically, Table 1 shows a comparison between the simulation results with Ω = 20,000 and those with Ω=500. We can observe from Table 1 that the average deviation for simulation results with Ω = 20,000 is only 1.56% (fairly close to the theoretical results), while that with Ω=500 is 8.35%.

### 5.2. Probability of Connection

#### 5.2.1. Impacts of Ambient Environment

**Figure 5:**
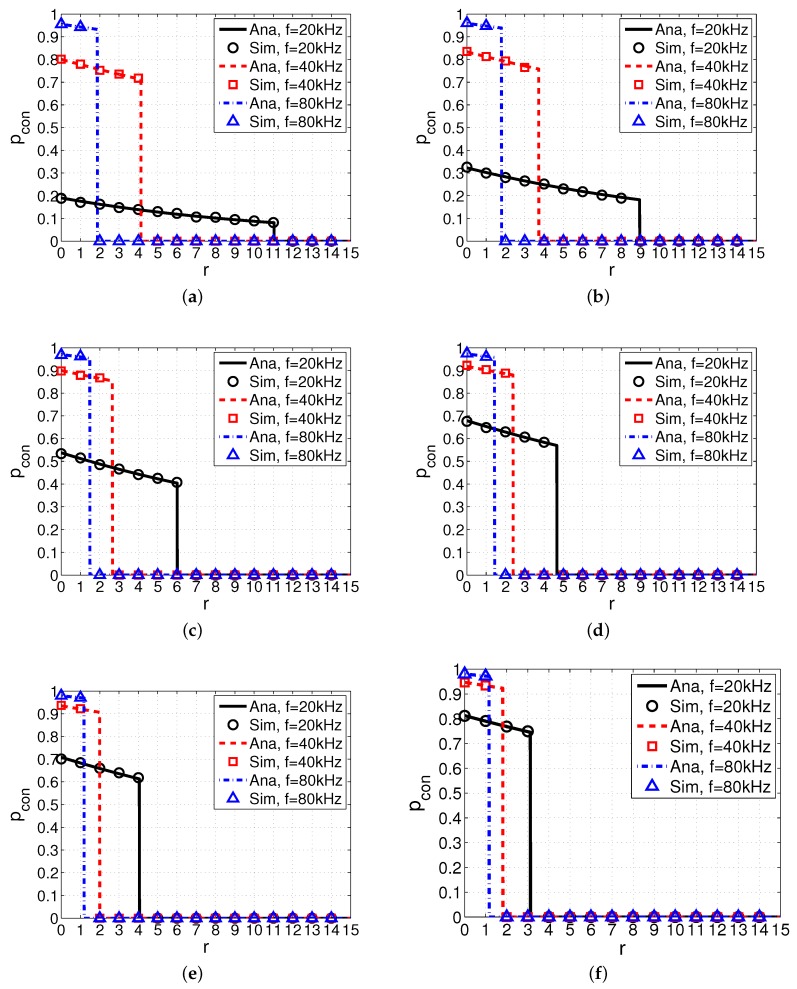
Probability of connection $p_{con}$ versus distance *r* with different spreading factor *k* and wind speed *w*. System parameters are set as $P_{p}=110$ dB, $P_{s}=100$ dB, $\lambda_{p}=0.003$, s=1, $\delta_{s}=20$ dB, and $\delta_{d}=20$ dB. (**a**) k=1,w=0 m/s; (**b**) k=2,w=0 m/s; (**c**) k=1,w=10 m/s; (**d**) k=2,w=10 m/s; (**e**) k=1,w=20 m/s; (**f**) k=2,w=20 m/s.

Figure 5 shows the results of the probability of connection $p_{con}$ versus distance *r* with different spreading factor *k* and wind speed *w*, where curves represent analytical results and markers represent simulation results. We can see that there is an excellent agreement between analytical results and simulation results, indicating that our analytical model is accurate. It is worth noting that $p_{con}$ drops to 0 when distance *r* reaches the maximum communication range $r_{\max}$, matching the aforementioned results in Equations (12) and (13).

Firstly, we make a horizontal comparison of $p_{con}$ with different values of *k* and identical values of *w*. *We observe that the increment of k results in the higher $p_{con}$ within the maximum communication range $r_{\max}$*. For example, when aligning Figure 5a and Figure 5b together, we can find that $p_{con}$ with k=2 is larger than that with k=1 at the same frequency. This is because larger spreading factor *k* results in higher attenuation and smaller value of $r_{d}$, consequently leading to higher spectrum availability. Moreover, *we also observe that the maximum communication range $r_{\max}$ with larger k is also shorter than that with smaller k*. Take Figure 5a,b as an example again. The maximum communication range $r_{\max}$ with k=2 and f=20 kHz is 9 km, smaller than that with k=1 and the same frequency (i.e., 11 km).

Secondly, we make a vertical comparison of $p_{con}$ with identical values of *k* and different values of *w*. *We observe that the increment of w leads to the higher* $p_{con}$ *within the maximum communication range* $r_{\max}$. Take Figure 5c,e as an example. We can see that $p_{con}$ with w=20 m/s is larger than that with w=10 m/s at the same frequency (e.g., f=20 kHz). This is because the larger value of *w* results in the smaller value of $r_{d}$, consequently leading to the higher spectrum availability. *Another observation is that the maximum communication range $r_{\max}$ with larger w is also shorter than that with smaller w*. For example, when aligning Figure 5c and Figure 5e together, $r_{\max}$ with w=20 m/s and f=20 kHz is 4 km (i.e., smaller than that with w=10 m/s and the same frequency).

Thirdly, we make a comparison of $p_{con}$ with different values of frequency *f*. *We observe that when frequency f increases, $p_{con}$ within $r_{\max}$ also increases*. Take Figure 5a as an example again. When *f* increases from 20 kHz to 80 kHz, though $r_{\max}$ becomes shorter, $p_{con}$ within $r_{\max}$ increases. This phenomenon may be owing to the fact that with a higher frequency, SUs have a shorter communication range, resulting in a higher spectrum availability and consequently enhancing the connectivity. This result implies that a proper frequency is necessary to ensure a higher probability of connection with the given communication range of SUs.

In summary, we observe that frequency *f* and ambient environment factors such as spreading factor *k* and wind speed *w* have a significant influence on the link connectivity of SUs in UCANs.

#### 5.2.2. Impacts of PUs

**Figure 6:**
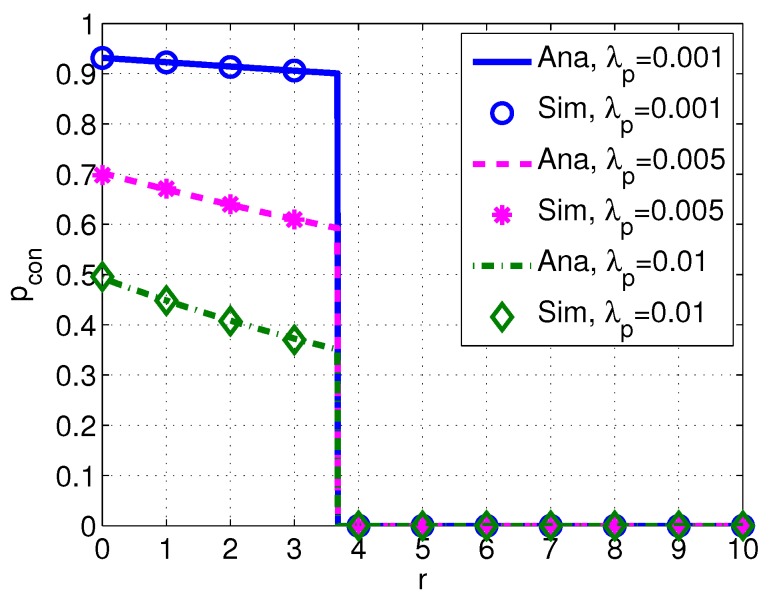
Probability of connection $p_{con}$ versus distance *r* with different intensity of PUs $\lambda_{p}$. System parameters are set as k=1, w=10 m/s, f=30 kHz, $P_{p}=110$ dB, $P_{s}=100$ dB, s=1, $\delta_{s}=20$ dB and $\delta_{d}=20$ dB.

Figure 6 shows the probability of connection $p_{con}$ versus distance *r* with different intensity of PUs $\lambda_{p}$. *We observe that $p_{con}$ decreases with the increased intensity $\lambda_{p}$*. This may owe to the fact that the higher intensity of PUs leads to the lower spectrum availability of SUs.

**Figure 7:**
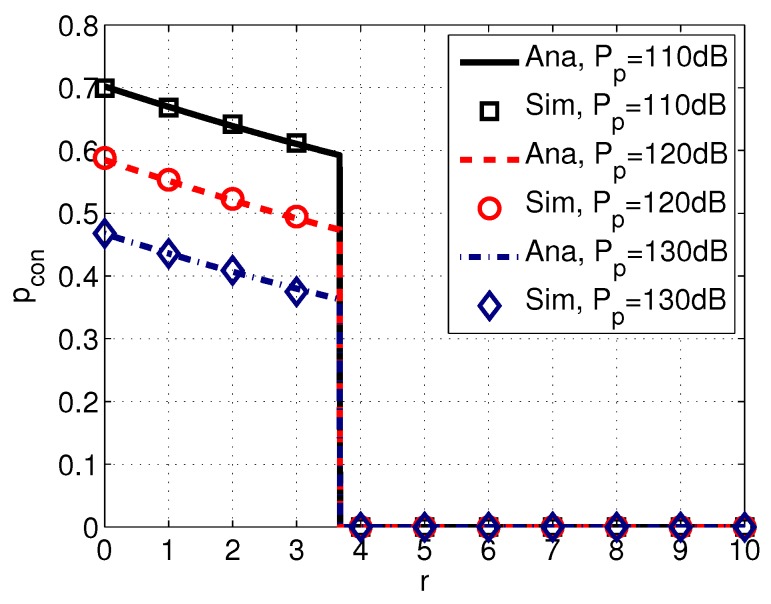
Probability of connection $p_{con}$ versus distance *r* with different transmission power of PUs $P_{p}$. System parameters are set as k=1, w=10 m/s, $\lambda_{p}=0.005$, f=30 kHz, $P_{s}=100$ dB, s=1, $\delta_{s}=20$ dB, and $\delta_{d}=20$ dB.

Figure 7 shows the probability of connection $p_{con}$ with different values of the transmission power of PUs $P_{p}$. *We observe that $p_{con}$ decreases with the increased transmission power $P_{p}$.* This is because PUs with higher $P_{p}$ can cause higher interference to SUs (in other words, they are more easily detected by SUs according to the detect and avoid protocol), and consequently SUs have less chance to transmit.

### 5.3. Probability of Coverage

**Figure 8:**
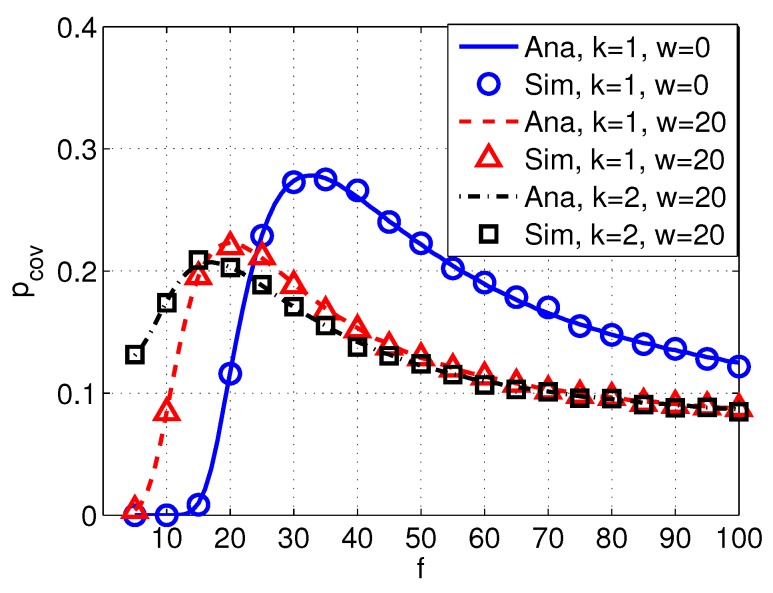
Coverage $p_{cov}$ versus frequency *f* with different spreading factor *k* and wind speed *w*. System parameters are set as $R_{d}=12$ km, $P_{p}=110$ dB, $P_{s}=100$ dB, $\lambda_{p}=0.003$, s=1, $\delta_{s}=20$ dB, and $\delta_{d}=20$ dB.

Figure 8 shows the probability of coverage $p_{cov}$ versus frequency *f* with different values of spread factor *k* and wind factor *w*. We can see that the probability of coverage varies with different acoustic frequencies. In particular, $p_{cov}$ increases with the increased frequency when frequency is relatively low. After $p_{cov}$ reaches the peak, $p_{cov}$ starts to decrease with the increased frequency. Moreover, the peak values of $p_{cov}$ decrease with the increased factors of *k* and *w*.

**Figure 9:**
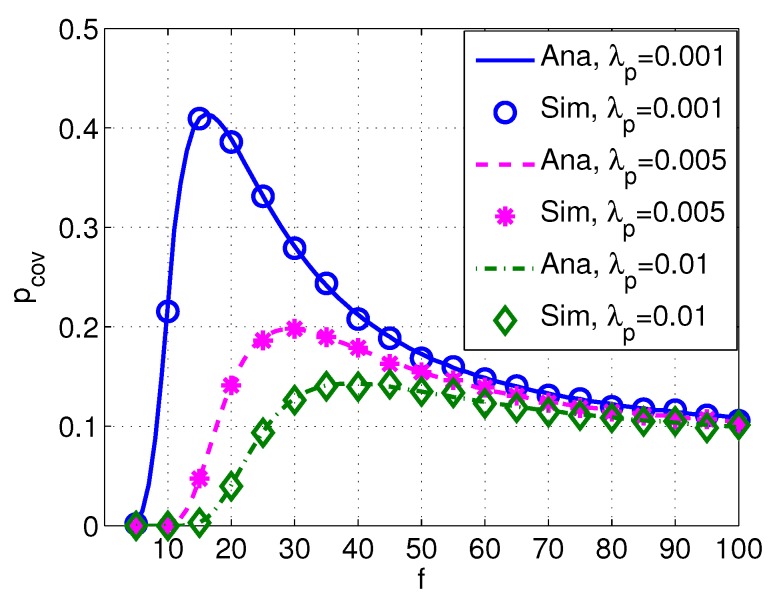
Coverage $p_{cov}$ versus frequency *f* with different $\lambda_{p}$. System parameters are set as $R_{d}=12$ km, $P_{p}=110$ dB, $P_{s}=100$ dB, s=1, k=1, w=10 m/s, $\delta_{s}=20$ dB, and $\delta_{d}=20$ dB.

**Figure 10:**
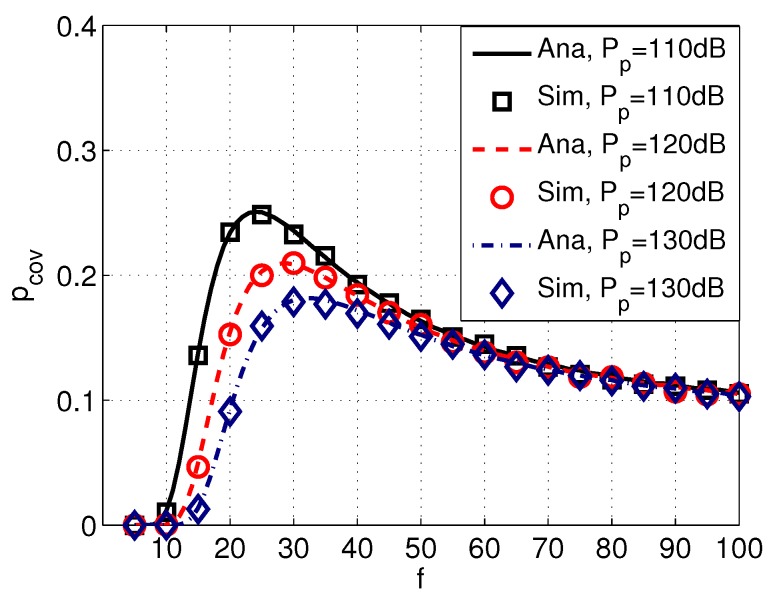
Coverage $p_{cov}$ versus frequency *f* with different power of PUs $P_{p}$. System parameters are set as $R_{d}=12$ km, $P_{s}=100$ dB, $\lambda_{p}=0.003$, k=1, w=10 m/s, s=1, $\delta_{s}=20$ dB, and $\delta_{d}=20$ dB.

Figure 9 shows the probability of coverage $p_{cov}$ versus frequency *f* with different values of intensity of PUs $\lambda_{p}$, and Figure 10 shows the probability of coverage $p_{cov}$ versus frequency *f* with different transmission power of PUs $P_{p}$. It can be seen from Figure 9 and Figure 10 that $p_{cov}$ decreases with a higher $\lambda_{p}$ or $P_{p}$, implying that the probability of coverage of SUs are significantly affected by PUs. Moreover, the peak value of $p_{cov}$ also varies with different values of $\lambda_{p}$ and $P_{p}$.

### 5.4. Discussion and Future Works

The above results show that both the link connectivity and probability of SUs in UCANs heavily depend on acoustic signal frequency, underwater environment factors, and the activities of PUs. Therefore, when system parameters as well as underwater environment are given, our analysis (i.e., $p_{con}$ and $p_{cov}$) can be used to find a proper signal frequency to acquire the optimal values for the link connectivity and the probability of coverage. Meanwhile, our results also offer an implication on optimizing the network throughput of SUs in UCANs. For example, if we assume SU pairs (i.e., both ST and SR) follow HPPP with intensity $\lambda_{s}$ in our UCAN model, the spatial throughput of SUs can be expressed by $p_{con}\lambda_{s}\log\left(1+\delta_{s}\right)$ (the spatial throughput is defined in [33]. We observe that the throughput of SUs is proportional to $p_{con}$.

In addition to the analysis of QoS metrics of UCANs, there are many interesting topics in UCANs. For example, how to design effective Medium Access Control (MAC) protocols, considering spectrum constraint [4,7,11,13]. Another issue is to design effective and efficient routing schemes [34] while considering the spectrum constraint. Our study presented in this paper has paved the way for the solutions to these research problems. For example, we can obtain the whole network topology once the link connectivity of each SU pair is obtained and we can then design the routing schemes based on the available topology.

## 6. Conclusions

In this paper, we investigate the link connectivity and the probability of coverage of secondary users in underwater cognitive acoustic networks (UCANs). In particular, we propose an analytical model to analyze the above QoS metrics of SUs in UCANs. This model takes both topological connectivity and spectrum availability into account. The extensive simulations validate the accuracy of our model. From the results, we observe that both the link connectivity and the probability of coverage of SUs in UCANs depend on frequency *f*, spreading factor *k*, and wind speed *w*. Meanwhile, both the link connectivity and the probability of coverage of SUs in UCANs are also significantly influenced by the activities of PUs (in terms of the intensity of PUs and transmission power of PUs). 

## Figures and Tables

**Table 1:** Deviation of $p_{con}^{s}$ between simulation results (with different values of Ω) and analytical results. System parameters are set as f=20 kHz, $P_{p}=110$ dB, $P_{s}=100$ dB, $\lambda_{p}=0.003$, k=1, w=0 m/s, s=1, $\delta_{s}=20$ dB, and $\delta_{d}=20$ dB.

	Analytical Value	Simulation Value with Ω = 500	Simulation Value with Ω = 20,000
*r* = 1 km	0.1758	0.1520 (13.53%)	0.1726 (0.18%)
*r* = 2 km	0.1624	0.1640 (0.99%)	0.1612 (0.74%)
*r* = 3 km	0.1500	0.1560 (4.00%)	0.1454 (3.07%)
*r* = 4 km	0.1386	0.1180 (14.86%)	0.1417 (2.24%)
Average deviation		8.35%	**1.56%**

## Appendix A. Attenuation

The attenuation underwater acoustic networks can be expressed as follows:

(A1) $$A\left(r,f\right)=r^{k}\alpha {\left(f\right)}^{r},$$

where *r* is the distance between a transmitter and a receiver, α(f) is an absorption coefficient of frequency *f*, *k* is a spreading factor (ranging from 1 to 2). Note that the different values of *k* describe the different scenarios of geometry of propagation: k=1 for cylindrical spreading; k=2 for spherical spreading.

Then, Equation (A1) can be expressed in dB as follows:

(A2) 10logA(r,f)=k·10logr+r·10logα(f).

Generally, if the frequency *f* is above a few hundred Hz, the absorption coefficient 10logα(f) in dB/km for *f* in kHz can be expressed as follows [21]:

(A3) $$\begin{array}{c} 10\log\alpha \left(f\right)=0.11\cdot \frac{f^{2}}{1+f^{2}}+44\frac{f^{2}}{4100+f^{2}}\\ +2.75\cdot 10^{-4}f^{2}+0.003. \end{array}$$

## Appendix B. Ambient Noise

Usually, the ambient noise in underwater environments is modeled by four kinds of sources: turbulence, shipping activities, waves, and thermal noise [22]. Specifically, the noise caused by shipping activities depends on activity factor *s* (ranging from 0 to 1). The noise of waves, brought by wind, can be expressed as a function of wind speed *w* (m/s). Then, the formula between power spectral density of the four noise sources and frequency in kHz in dB re μ Pa per Hz are given by [29] as follows:

(A4) $$\begin{array}{c} 10\log N_{t}\left(f\right)=17-30\log\left(f\right),\\ 10\log N_{s}\left(f\right)=40+20\left(s-0.5\right)+26\log\left(f\right)-60\log\left(f+0.03\right),\\ 10\log N_{w}\left(f\right)=50+7.5w^{1/2}+20\log\left(f\right)-40\log\left(f+0.4\right),\\ 10\log N_{th}\left(f\right)=-15+20\log\left(f\right), \end{array}$$

where $N_{t}\left(f\right)$, $N_{s}\left(f\right)$, $N_{w}\left(f\right)$, and $N_{th}\left(f\right)$ express the noise produced by turbulence, shipping activity, wind, and thermal, respectively.

Then the total noise can be expressed as:

(A5) $$10\log N\left(f\right)=10\log(N_{t}\left(f\right)+N_{s}\left(f\right)+N_{w}\left(f\right)+N_{th}\left(f\right)).$$
